# Plasma creatine concentration is associated with incident hypertension in a cohort enriched for the presence of high urinary albumin concentration: the Prevention of Renal and Vascular Endstage Disease study

**DOI:** 10.1097/HJH.0000000000002996

**Published:** 2021-08-09

**Authors:** Adrian Post, Daan Kremer, J. Casper Swarte, Sara Sokooti, Fabian A. Vogelpohl, Dion Groothof, Ido.P. Kema, Erwin Garcia, Margery A. Connelly, Theo Wallimann, Robin P.F. Dullaart, Casper F.M. Franssen, Stephan J.L. Bakker

**Affiliations:** a Department of Internal Medicine; b Department of Laboratory Medicine, University Medical Center Groningen, University of Groningen, Groningen, the Netherlands; c Laboratory Corporation of America Holdings (Labcorp), Morrisville, North Carolina, USA; d Department of Biology, ETH Zurich, Zurich, Switzerland

**Keywords:** adults, creatine, hypertension

## Abstract

: Hypertension is a major risk factor for cardiovascular disease, kidney disease, and premature death. Increased levels of creatine kinase are associated with development of hypertension. However, it is unknown if creatine, a substrate of CK, is associated with the development of hypertension. We therefore, aimed to investigate the association between plasma creatine concentration and incident hypertension.

**Methods:**

We measured fasting plasma creatine concentrations by nuclear magnetic resonance spectroscopy in participants of the population-based PREVEND study. The study outcome was incident hypertension, defined as either a SBP of at least 140 mmHg, a DBP of at least 90 mmHg, or the new usage of antihypertensive drugs. Participants with hypertension at baseline were excluded.

**Results:**

We included 3135 participants (46% men) aged 49 ± 10 years. Mean plasma creatine concentrations were 36.2 ± 17.5 μmol/l, with higher concentrations in women than in men (42.2 ± 17.6 versus 29.2 ± 17.6 μmol/l; *P* < 0.001). During a median of 7.1 [interquartile range: 3.6–7.6] years of follow-up, 927 participants developed incident hypertension. Higher plasma creatine concentrations were associated with an increased risk of incident hypertension [HR per doubling of plasma creatine: 1.21 (95% confidence interval: 1.10–1.34); *P* < 0.001], which remained significant after adjustment for potential confounders. Sex-stratified analyses demonstrated higher plasma creatine that was independently associated with an increased risk of incident hypertension in men [hazard ratio: 1.26 (95% CI 1.11–1.44); *P* < 0.001], but not in women (hazard ratio: 1.13 (95% CI 0.96–1.33); *P* = 0.14]. Causal pathway analyses demonstrate that the association was not explained by sodium or protein intake.

**Conclusion:**

Higher plasma creatine is associated with an increased risk of hypertension in men. Future studies are warranted to determine the underlying mechanisms.

## INTRODUCTION

Hypertension is a major risk factor for cardiovascular disease, chronic kidney disease, and premature death worldwide [1]. The universal biomarker for hypertension is blood pressure, defining the condition and guiding therapeutic approaches. None-the-less, novel biomarkers for hypertension are necessary to further unravel the pathophysiology of hypertension and to pave the way for novel therapeutic strategies [2]. The activity of the ATP-generating enzyme creatine kinase has been identified as a biomarker for hypertension, and since then multiple studies have confirmed creatine kinase as a biomarker for hypertension [3–7]. However, to date, no studies have explored whether circulating concentrations of the substrate of creatine kinase, creatine, are also related to hypertension in adults not using creatine supplementation. Creatine is a natural nitrogenous organic acid that is integral to cellular energy metabolism, which should be differentiated from creatinine. Creatine and creatinine are in a chemical equilibrium that favors conversion of creatine to creatinine under physiological conditions [8,9]. As creatinine is freely excreted, a continuous supply of creatine is required to replenish losses. Creatine can be synthesized endogenously but can also be ingested through the diet. In the diet, creatine is primarily found in meat and fish [10], which is reflected by vegetarians having lower muscle creatine levels than nonvegetarians [11,12]. The biochemical synthesis of creatine starts with the conversion of arginine and glycine into guanidinoacetate [13–17]. Guanidinoacetate is subsequently converted into creatine in the liver, after which it is released into the circulation [18].

In the current study, we aimed to determine the plasma creatine concentrations in a large population-based cohort, in order to prospectively investigate the association of plasma creatine with incident hypertension. Given the established sex differences in the rates of hypertension, and the fact that location of the creatine transporter gene resides on the X-chromosome [19–21], we also performed sex-stratified analyses. Additionally, we performed causal pathway analyses.

## METHODS

### Study design and participants

The Prevention of Renal and Vascular Endstage Disease (PREVEND) study has been approved by the medical ethics committee of the University Medical Center Groningen and was undertaken in accordance with the Declaration of Helsinki [22]. The PREVEND study prospectively investigates risk factors for microalbuminuria and the prevalence and consequences of microalbuminuria in otherwise healthy adults (28--75 years) in the city of Groningen (The Netherlands). The objectives and design have been described in detail elsewhere [23].

In short, during 1997 and 1998, all 85 421 inhabitants of the city of Groningen between the ages of 28 and 75 years were invited to participate in the study and were sent a one-page questionnaire regarding demographics, cardiovascular morbidity, use of medication, menstruation, and pregnancy along with a vial to collect a first morning void urine sample.

From this group, 30 890 participants had a urinary albumin concentration of less than 10 mg/l and 9966 participants had a urinary albumin concentration of ≥10 mg/l in their morning urine sample.

After exclusion of participants without written consent, participants with insulin usage and pregnant women, all participants with a urinary albumin concentration of at least 10 mg/l (*n* = 7768) together with a randomly selected control group with a urinary albumin concentration of less than 10 mg/l (*n* = 3395) were invited for further investigations in an outpatient clinic (total *n* = 11 163). In total, 8592 participants completed the total screening program in 1997–1998. A total of 6894 participants participated in the second screening round in 2001–2003, which was the baseline of the current study. We excluded 2380 participants with hypertension at baseline, 1488 participants with no data on plasma creatine concentration and 578 participants without follow-up data on the incidence of hypertension. After accounting for overlap, this led to exclusion of 3759 participants and a total of 3135 participants for the current study. All participants who participated in the current study provided written informed consent. Follow-up was until 1 January 2011. Detailed information on the flow of participants through the study is provided in Figure S1.

### Clinical and laboratory measurements

Each screening constituted two visits to an outpatient clinic separated by 3 weeks. Self-administered questionnaires concerning demographics, cardiovascular and kidney disease history, smoking habits, and medication use were provided by all participants prior to the first visit. Information on medication use was combined with information from IADB.nl, a database containing information of prescribed medication in public pharmacies in The Netherlands since 1999 (http://www.iadb.nl/). Anatomic Therapeutic Chemical codes C10 was used to define lipid-lowering drugs [24]. Height and weight were measured with the participants standing without shoes and heavy outer garments. BMI was calculated by dividing weight in kilograms by height, in meters, squared. Baseline EDTA plasma samples were drawn from all participants between 0800 and 1000 h, after an instructed overnight fast, and aliquots of these samples were immediately stored at −80 °C until analysis. Fasting plasma glucose was measured by dry chemistry (Eastman Kodak, Rochester, New York, USA). Serum creatinine was measured with an enzymatic method on a Roche Modular analyzer, using reagents and calibrators from Roche (Roche Diagnostics, Mannheim, Germany). Total cholesterol, high-density lipoprotein (HDL)-cholesterol triglycerides, insulin, urinary sodium, urinary potassium, urinary urea, and serum cystatin C were measured using standard protocols, which have been described previously [25–28]. N-terminal pro-B-type natriuretic peptide (NT-proBNP) was assayed in plasma using a Elecsys 2010 analyzer. Plasma branched-chain amino acids (BCAA) were measured using a validated nuclear magnetic resonance spectroscopy assay (Labcorp, Morrisville, North Carolina, USA) [29]. Urinary sulfate was measured by means of a validated ion-exchange chromatography assay with conductivity detection (Metrohm, Herisau, Switzerland) [30]. Estimated glomerular filtration rate (eGFR) was calculated using the Chronic Kidney Disease Epidemiology Collaboration (CKD-EPI) combined creatinine–cystatin C equation [31]. Fractional reabsorption of sodium was defined as one minus the fractional excretion of sodium [30].

### Creatine assay

Creatine was measured using a Vantera nuclear magnetic resonance (NMR) Clinical Analyzer (Labcorp, Raleigh, North Carolina, USA), as previously described [32]. For the creatine assay, a plasma volume of 300 μl was required. Plasma samples were mixed (3 : 1 v/v) with citrate/phosphate buffer to adjust the pH to 5.3, in order to separate the creatine and creatinine on the proton NMR spectra, which overlap at physiological pH [33,34]. The creatine peak was quantified using a proprietary lineshape deconvolution by a nonnegative least squares fitting algorithm, which models the peak as Lorentzian and Gaussian lineshapes. The creatine signal amplitudes were multiplied with an empirical factor, determined from standard spiking, to convert it to concentration (μmol/l). Creatine results from the NMR quantification software agree well with routine enzymatic (creatinase)/spectrophotometry assay (*R*^2^ = 0.995, slope = 0.99, intercept = 12.2, *n* = 44). The intra-assay and inter-assay precision (expressed as percent coefficient of variation) for the NMR assay were 4.0 and 4.9%, respectively. Due to the requirement of a relatively large volume of plasma sample for performance of the assay, creatine concentration could not be assessed in all participants.

### Blood pressure measurement and incident hypertension

Participants were followed from the date of the baseline visit until end of follow-up. At both visits of each examination, blood pressure was assessed on the right arm in supine position, every minute for ten and eight minutes, respectively, with an automatic Dinamap XL Model 9300 series device [35,36]. The mean of the last two recordings from each visit was used. The procedure has been previously described [37]. Use of antihypertensive medications was ascertained by a questionnaire at each examination. Incident hypertension was defined as hypertension that occurred after baseline, specified as either a SBP of at least 140 mmHg, a DBP of at least 90 mmHg, or the newly recorded use of antihypertensive drugs. Antihypertensive medication use, for the definition of hypertension, included five second-level Anatomic Therapeutic Chemical codes [24]: C02 (antihypertensives, i.e. alpha-adrenergic blockers), C03 (diuretics), C07 (β-blockers), C08 (calcium channel blockers), and C09 (inhibitors acting on the renin--angiotensin system).

### Statistical analyses

Results were expressed as mean ± standard deviation (SD), median [interquartile range], or number (percentage) for normally distributed, skewed, and categorical data, respectively. For plasma creatine concentrations, despite a somewhat skewed distribution, we displayed the mean ± SD to facilitate comparisons with existing literature on creatine concentrations. A two-sided *P* value less than 0.05 was considered to indicate statistical significance. The distribution of plasma creatine concentrations in men and women are demonstrated using a dot-plot. Baseline characteristics are presented for the whole cohort and according to sex. Differences in baseline characteristics between men and women are tested using independent sample *t* test, Wilcoxon -Mann -Whitney U test, or chi-squared test. Multivariable linear regression was used to assess the association of plasma creatine concentrations with baseline variables. Regression models were adjusted for age and sex. For these analyses, regression coefficients were given as standardized beta values, thereby allowing for comparison of the strength of the associations of different variables. Analyses were performed for the total cohort as well as stratified according to sex. The assumption of normally distributed error terms was validated by inspection of Q--Q plots of the residuals.

Cox proportional hazards models were used to investigate the associations of plasma creatine concentrations with incident hypertension. Hazard ratios were computed per doubling of plasma creatine. The proportional hazards assumption was verified visually with plots of the scaled Schoenfeld residuals and was not violated in any of the models. Analyses were performed for the total cohort and stratified according to sex. Adjustments were made for a priori selected variables, including SBP, age, sex, BMI, eGFR, urinary albumin excretion, NT-proBNP, total cholesterol, HDL cholesterol, triglycerides, use of lipid-lowering drugs, alcohol intake and smoking status. Lastly, in separate causal pathway models, adjustments were made for 24-h urinary excretion of sodium, potassium, urea, and sulfate, plasma branched chain amino acids, type 2 diabetes, and plasma creatinine. Potential effect-modification of the association between plasma creatine and incident hypertension by sex was explored by including product terms to the SBP and sex-adjusted models.

To account for potential bias that could result from the exclusion of participants with missing values [38], multiple imputation using Fully Conditional Specification was performed using the ‘mice’ package to obtain five imputed data sets. The algorithm was run 30 iterations and convergence of the Markov chains was evaluated with trace plots of the mean and variance. To confirm that imputed values were biologically plausible, the distributions of the imputed values were visually investigated and compared with the distribution of the observed values. Analyses were performed in each of the data sets and results were pooled using Rubin's rules [38,39]. Apart from the baseline table and unless otherwise stated, analyses were performed using imputed data sets. To visualize the continuous associations of plasma creatine with incident hypertension, plasma creatine, as a continuous variable, was plotted against the risk of incident hypertension in men and women separately.

Sensitivity analyses were conducted to evaluate the robustness of the findings, wherein any potential bias caused by outliers in plasma creatine was accounted for by excluding participants with plasma creatine values in the highest and lowest 2.5 percentiles. Given the strong predictive value of baseline blood pressure, we performed an additional sensitivity analysis in which we excluded participants with a SBP between 130 and 139 mmHg and/or a DBP between 80 and 90 mmHg. Furthermore, to account for possible genetic aspects of hypertension, we performed a sensitivity analysis in which we excluded participants with a parent diagnosed with hypertension. Also, we performed a comparison in baseline characteristics between participants with and without plasma creatine concentrations available. Statistical analyses were performed with R version 3.6.2 (Vienna, Austria; http://cran.r-project.org/).

## RESULTS

### Baseline characteristics

**FIGURE 1:**
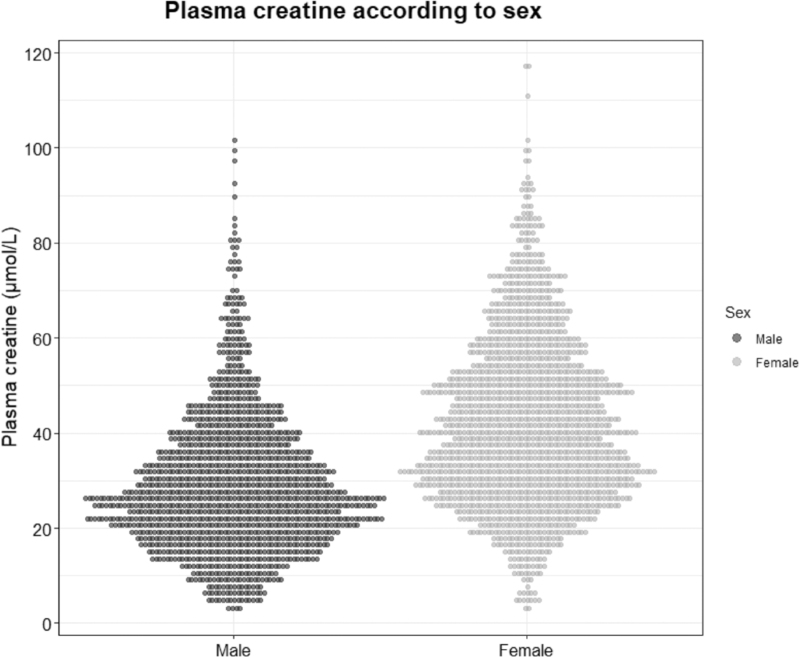
Mirror histogram displaying the sex-based differences in plasma creatine. Mean plasma creatine is 27.4 ± 14.0 μmol/l in men and 32.5 ± 14.8 μmol/l in women. *P* for difference less than 0.001.

A total of 3135 participants without hypertension at baseline were included. The mean age was 49 ± 10 years and 1441 (46%) were men. The baseline plasma creatine concentration was 36.2 ± 17.5 μmol/l. Plasma creatine concentrations were substantially higher in women than in men (42.2 ± 17.6 μmol/l versus 29.2 ± 17.6 μmol/l; *P* < 0.001). Unlike creatine, plasma creatinine concentrations were higher in men than in women (78 ± 12 μmol/l versus 64 ± 9 μmol/l; *P* < 0.001). The distributions of plasma creatine concentrations in men and women are shown in Fig. 1. Baseline characteristics of the study participants stratified according to sex are shown in Table 1. Compared with women, men had higher age, BMI, waist circumference, SBP, DBP, pulse rate, plasma creatinine, eGFR, alcohol intake, triglycerides, plasma glucose, and plasma insulin, as well as higher urinary albumin, sodium, potassium, urea, and sulfate excretion, and plasma BCAA (all *P* < 0.05). Women had a higher prevalence of parental hypertension as well as higher NT-proBNP, and HDL cholesterol compared with men (all *P* < 0.05).

**TABLE 1:** Baseline clinical and laboratory characteristics in 3135 participants of the PREVEND study without hypertension at baseline

Variables	Total cohort (*n *= 3135)	Men (*n* = 1441)	Women (*n* = 1694)	*P* value
Plasma creatine (μmol/l)	36.2 ± 17.5	29.2 ± 14.5	42.2 ± 17.6	<0.001
Age (years)	49.3 ± 10.2	49.8 ± 10.7	48.9 ± 9.7	0.02
BMI (kg/m^2^)	25.7 ± 3.8	25.9 ± 3.3	25.6 ± 4.2	0.03
Waist circumference (cm)	89 ± 11	94 ± 10	85 ± 11	<0.001
SBP (mmHg)	118 ± 11	122 ± 10	114 ± 11	<0.001
DBP (mmHg)	70 ± 7	73 ± 7	68 ± 7	<0.001
Pulse rate (bpm)	89 ± 12	94 ± 10	85 ± 11	<0.001
NT-proBNP (ng/l)	35 [18–62]	22 [11–40]	48 [29–77]	<0.001
Plasma creatinine (μmol/l)	71 ± 13	78 ± 12	64 ± 9	<0.001
eGFR (ml/min per 1.73 m^2^)	97 ± 14	98 ± 15	96 ± 14	0.001
Parental history of hypertension [*n* (%)]	952 (33)	381 (29)	571 (36)	<0.001
Current smoker [*n* (%)]	930 (30)	423 (29)	507 (30)	0.75
Consumption of alcohol [*n* (%)]	2240 (78)	1220 (84)	1220 (72)	<0.001
Usage of lipid-lowering drugs [*n* (%)]	94 (4)	47 (4)	47 (3)	0.24
Total cholesterol (mmol/l)	5.36 ± 1.03	5.39 ± 1.01	5.34 ± 1.04	0.19
HDL cholesterol (mmol/l)	1.28 ± 0.30	1.13 ± 0.24	1.40 ± 0.30	<0.001
Triglycerides (mmol/l)	1.0 [0.8–1.5]	1.2 [0.8–1.7]	1.0 [0.7–1.3]	<0.001
Glucose (mmol/l)	4.7 [4.4–5.1]	4.8 [4.5–5.2]	4.6 [4.3–5.0]	<0.001
Insulin (mU/l)	7.3 [5.3–10.5]	7.7 [5.5–11.3]	7.1 [5.2–9.8]	<0.001
Plasma branched chain amino acids (μmol/l)	371 ± 70	410 ± 64	338 ± 56	<0.001
Urinary albumin excretion (mg/24 h)	7.7 [5.8–12.3]	8.4 [6.1–14.1]	7.3 [5.6–11.0]	<0.001
Urinary sodium excretion (mmol/24 h)	145 ± 55	163 ± 60	129 ± 45	<0.001
Urinary potassium excretion (mmol/24 h)	70 ± 22	77 ± 23	65 ± 20	<0.001
Urinary urea excretion (mmol/24 h)	366 ± 113	411 ± 119	328 ± 93	<0.001
Urinary sulfate excretion (mmol/24 h)	16 [12–20]	18 [14–23]	14 [11–18]	<0.001
Sodium reabsorption (%)	99.42 ± 0.19	99.39 ± 0.19	99.42 ± 0.18	<0.001

Results were expressed as mean ± standard deviation (SD), median [interquartile range], or number (percentage) for normally distributed, skewed, and categorical data, respectively.

### Linear regression analyses

**FIGURE 2:**
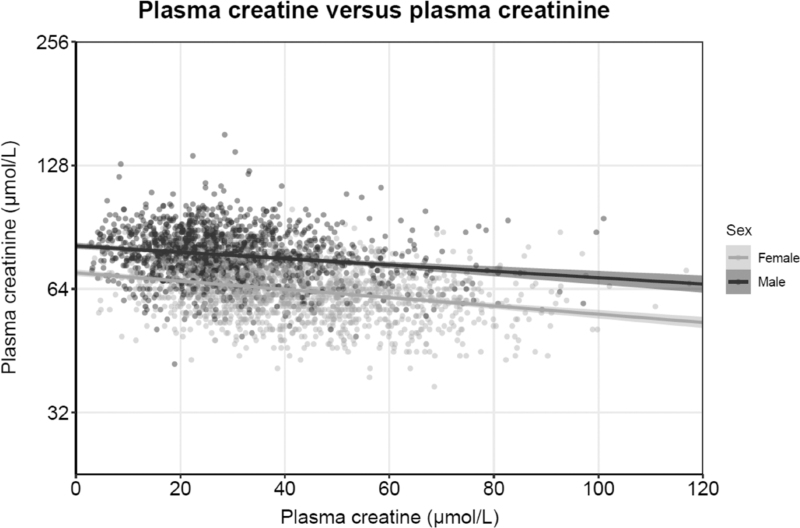
A scatterplot of the association between plasma creatinine and plasma creatine.

Multivariable linear regression analyses are shown in Table 2. Baseline plasma creatine was positively associated with age, BMI, waist circumference, eGFR, current smoking status, total cholesterol, triglycerides, glucose, insulin (all *P* < 0.05), all with higher standardized beta values in men than in women. In contrast, plasma creatine was more strongly associated with pulse rate and plasma BCAA in women than in men. Baseline creatine was positively associated with SBP, DBP and alcohol intake in men but not in women. Plasma creatine was inversely associated with plasma creatinine, NT-proBNP and HDL-cholesterol in both men and women (all *P* < 0.05). The inverse association of plasma creatine versus plasma creatinine concentrations is shown in Fig. 2.

**TABLE 2:** Multivariable linear regression analyses of plasma creatine with selected variables at baseline

Dependent variables	Total cohort (*n* = 3135)		Men (*n* = 1441)		Women (*n* = 1694)	
Age (years)	0.17 [0.13; 0.21]	<0.001	0.20 [0.14; 0.27]	<0.001	0.15 [0.11; 0.19]	<0.001
BMI (kg/m^2^)	0.19 [0.16; 0.23]	<0.001	0.23 [0.18; 0.28]	<0.001	0.17 [0.12; 0.22]	<0.001
Waist circumference (cm)	0.01 [−0.01; 0.03]	0.26	0.03 [0.01; 0.06]	0.03	−0.01 [−0.03; − 0.02]	0.88
SBP (mmHg)	0.01 [−0.02; 0.05]	0.42	0.01 [−0.04; 0.06]	0.63	0.01 [−0.03; 0.05]	0.67
DBP (mmHg)	0.07 [0.04; 0.11]	<0.001	0.12 [0.06; 0.17]	<0.001	0.04 [−0.01; 0.09]	0.06
Pulse rate (bpm)	0.08 [0.04; 0.11]	<0.001	0.07 [0.01; 0.14]	0.04	0.08 [0.03; 0.12]	0.001
NT-proBNP^a^ (ng/l)	−0.11 [−0.14; −0.07]	<0.001	−0.14 [−0.20; −0.07]	<0.001	−0.09 [−0.13; −0.05]	<0.001
Plasma creatinine^a^ (μmol/l)	−0.23 [−0.26; −0.19]	<0.001	−0.22 [−0.28; −0.16]	<0.001	−0.24 [−0.28; −0.20]	<0.001
eGFR (ml/min per 1.73m^2^)	0.19 [0.16; 0.22]	<0.001	0.17 [0.12; 0.23]	<0.001	0.20 [0.16; 0.24]	<0.001
Parental history of hypertension [*n* (%)]	0.01 [-0.01; 0.03]	0.20	0.01 [−0.02; 0.05]	0.50	0.01 [−0.01; 0.04]	0.34
Current smoker [*n* (%)]	0.07 [0.05; 0.09]	<0.001	0.12 [0.09; 0.15]	<0.001	0.05 [0.02; 0.07]	<0.001
Consumption of alcohol [*n* (%)]	−0.01 [−0.02; 0.01]	0.89	0.01 [−0.01; 0.04]	0.23	−0.01 [−0.03; 0.01]	0.32
Usage of lipid-lowering drugs [*n* (%)]	0.01 [−0.01; 0.01]	0.22	−0.01 [−0.02; 0.01]	0.45	0.01 [0.01; 0.02]	0.03
Total cholesterol (mmol/l)	0.08 [0.04; 0.12]	<0.001	0.08 [0.02; 0.14]	0.01	0.07 [0.03; 0.12]	0.001
HDL cholesterol (mmol/l)	−0.07 [−0.10; −0.03]	<0.001	−0.11 [−0.15; −0.06]	<0.001	−0.04 [−0.09; 0.01]	0.06
Triglycerides^a^ (mmol/l)	0.07 [0.04; 0.11]	<0.001	0.13 [0.06; 0.19]	<0.001	0.04 [−0.01; 0.08]	0.08
Glucose^a^ (mmol/l)	0.06 [0.02; 0.10]	0.002	0.10 [0.04; 0.16]	0.002	0.04 [−0.01; 0.08]	0.12
Insulin^a^ (mU/l)	0.05 [0.02; 0.09]	0.001	0.06 [0.01; 0.12]	0.03	0.05 [0.01; 0.09]	0.02
Plasma branched chain amino acids (μmol/l)	0.09 [0.06; 0.12]	<0.001	0.04 [−0.01; 0.10]	0.12	0.11 [0.07; 0.14]	<0.001
Urinary albumin excretion^a^ (mg/24 h)	0.10 [0.06; 0.14]	<0.001	0.16 [0.09; 0.22]	<0.001	0.07 [0.02; 0.11]	0.005
Urinary sodium excretion (mmol/24 h)	0.10 [0.07; 0.14]	<0.001	0.14 [0.08; 0.21]	<0.001	0.07 [0.04; 0.11]	<0.001
Urinary potassium excretion (mmol/24 h)	0.02 [−0.02; 0.05]	0.37	−0.01 [−0.08; 0.06]	0.79	0.03 [−0.01; 0.07]	0.17
Urinary urea excretion (mmol/24 h)	0.14 [0.10; 0.17]	<0.001	0.16 [0.09; 0.22]	<0.001	0.12 [0.08; 0.16]	<0.001
Urinary sulfate excretion^a^ (mmol/24 h)	0.13 [0.09; 0.17]	<0.001	0.12 [0.06; 0.18]	<0.001	0.13 [0.09; 0.18]	<0.001
Sodium reabsorption (%)	0.02 [−0.02; 0.06]	0.35	−0.01 [−0.08; 0.05]	0.70	0.04 [−0.01; 0.09]	0.11

All models are adjusted for age, sex, and BMI. Unless otherwise stated, analyses are performed on imputed datasets.

a Log_2_ transformed for analyses.

### Longitudinal analyses in the whole cohort

A total of 927 out of 3135 participants (30%) developed incident hypertension during a follow-up of 7.1 [3.6–7.6] years. Participants who developed incident hypertension during follow-up had significantly higher plasma creatine concentrations compared with those who did not develop hypertension (38.8 ± 17.5 versus 35.1 ± 17.3 μmol/l; *P* < 0.001). An overview of Cox regression analyses is shown in Table 3. For every doubling of plasma creatine, the sex and SBP-adjusted hazard ratio for incident hypertension was 1.39 (95% CI 1.26–1.53; *P* < 0.001). After additional adjustment for age, BMI, eGFR, and urinary albumin excretion, the hazard ratio changed to 1.21 (95% CI 1.10–1.34; *P* < 0.001). Further adjustment for other potential confounders, including parental history of hypertension, NT-proBNP, total cholesterol, HDL-cholesterol, triglycerides, usage of lipid-lowering drugs, smoking status, and alcohol intake did not materially change the association. Causal pathway analyses investigating the effect of 24-h urinary excretion of sodium, potassium, urea and sulfate, plasma BCAA, type 2 diabetes, and plasma creatinine on the association between plasma creatine concentrations and incident hypertension are shown in Table 4. Adjustment for 24-h urinary excretion of sodium, potassium, urea, and sulfate did not materially change the association between plasma creatine concentration and incident hypertension. Neither did adjustment for plasma BCAA, type 2 diabetes, or plasma creatinine.

**TABLE 3:** Longitudinal associations of plasma creatine with the risk of incident hypertension

	Total cohort		Men		Women	
	Per doubling		Per doubling		Per doubling	
	HR [95% CI]	*P* value	HR [95% CI]	*P* value	HR [95% CI]	*P* value
Model 1	1.39 [1.26–1.53]	<0.001	1.45 [1.28–1.64]	<0.001	1.29 [1.11–1.50]	0.001
Model 2	1.23 [1.11–1.35]	<0.001	1.26 [1.11–1.43]	<0.001	1.16 [0.99–1.36]	0.06
Model 3	1.21 [1.10–1.34]	<0.001	1.26 [1.11–1.44]	<0.001	1.13 [0.96–1.33]	0.14
Model 4	1.22 [1.10–1.35]	<0.001	1.27 [1.12–1.45]	<0.001	1.13 [0.96–1.33]	0.14
Model 5	1.28 [1.10–1.48]	0.001	1.24 [1.08–1.41]	0.002	1.16 [0.95–1.31]	0.19
Model 6	1.17 [1.06–1.30]	0.002	1.20 [1.05–1.37]	0.009	1.10 [0.94–1.31]	0.20
Participants	3135		1441		1694	
Events	927		436		491	

Model 1: adjusted for sex (only for total cohort) and SBP. Model 2: as model 1, additionally adjusted for age and BMI. Model 3; as model 2, additionally adjusted for eGFR and urinary albumin excretion. Model 4, as model 3, additionally adjusted for parental history of hypertension and NT-proBNP. Model 5, as model 3, additionally adjusted for total cholesterol, HDL cholesterol, triglycerides, and usage of lipid-lowering drugs. Model 6, as model 3, additionally adjusted for smoking and alcohol intake.

**TABLE 4:** Causal pathway analyses of the association between plasma creatine concentration and incident hypertension

	Total cohort		Men		Women	
	Per doubling		Per doubling		Per doubling	
	HR [95% CI]	*P* value	HR [95% CI]	*P* value	HR [95% CI]	*P* value
Base model	1.21 [1.10–1.34]	<0.001	1.26 [1.11–1.44]	<0.001	1.13 [0.96–1.33]	0.14
Adjusted for 24-h urinary sodium excretion	1.23 [1.11–1.36]	<0.001	1.27 [1.11–1.45]	<0.001	1.13 [0.96–1.34]	0.14
Adjusted for 24-h urinary potassium excretion	1.22 [1.10–1.35]	<0.001	1.27 [1.12–1.45]	<0.001	1.13 [0.96–1.33]	0.14
Adjusted for 24-h urinary urea excretion	1.24 [1.12–1.37]	<0.001	1.29 [1.13–1.47]	<0.001	1.15 [0.98–1.36]	0.09
Adjusted for 24-h urinary sulfate excretion	1.21 [1.09–1.34]	<0.001	1.28 [1.12–1.46]	<0.001	1.13 [0.96–1.32]	0.15
Adjusted for plasma BCAA	1.21 [1.09–1.33]	<0.001	1.26 [1.11–1.44]	<0.001	1.11 [0.95–1.31]	0.19
Adjusted for type 2 diabetes	1.22 [1.10–1.35]	<0.001	1.27 [1.11–1.45]	<0.001	1.13 [0.96–1.33]	0.13
Adjusted for creatinine	1.20 [1.08–1.33]	<0.001	1.26 [1.10–1.43]	<0.001	1.10 [0.93–1.29]	0.26

Base model is adjusted for sex (total cohort only), SBP, age, BMI, eGFR and urinary albumin excretion. BCAA, branched-chain amino acids; CI, confidence interval; HR, hazard ratio.

### Sex-stratified prospective analyses

**FIGURE 3:**
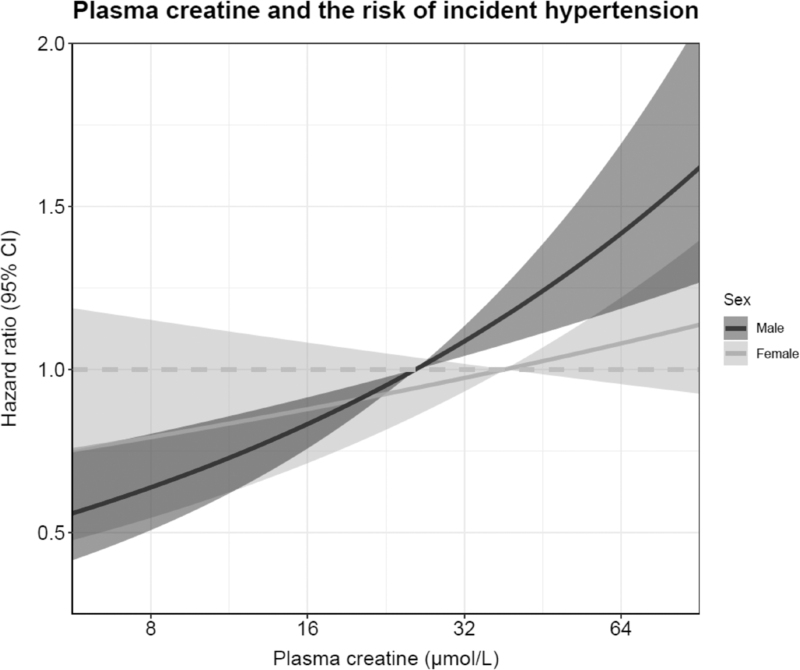
Graphical representation of the association of plasma creatine and the risk of incident hypertension in men and women. The lines show the adjusted hazard ratio (HR) and the gray area corresponds to the 95% pointwise confidence interval (CI). The analyses were stratified for sex and adjusted for SBP, age, BMI, eGFR and urinary albumin excretion. *P* effect are <0.001 and 0.22 in men and women, respectively. eGFR, estimated glomerular filtration rate.

Among 1441 men and 1694 women, 436 (30%) men and 491 (29%) women developed incident hypertension. The association between plasma creatine and incident hypertension was subject to substantial effect-modification by sex, as evidenced by a significant interaction term (*P* = 0.04). Men who developed incident hypertension during follow-up had significantly higher plasma creatine concentrations compared with those who did not develop incident hypertension (32.5 ± 14.8 versus 27.4 ± 14.0 μmol/l; *P* < 0.001). Women who developed incident hypertension during follow-up also had significantly higher plasma creatine concentrations to those who did not develop incident hypertension (45.9 ± 17.6 versus 40.9 ± 17.4 μmol/l; *P* < 0.001). An overview of Cox regression analyses in men and women is shown in Table 3. In Cox regression analyses in men, plasma creatine was strongly associated with the risk of incident hypertension. For every doubling of plasma creatine concentration, the SBP-adjusted hazard ratio for incident hypertension in men was 1.45 [1.28–1.64] (*P* < 0.001). After additional adjustment for age, BMI, eGFR, and urinary albumin excretion the hazard ratio changed to 1.26 [1.11–1.44] (*P* < 0.001). Further adjustment for the other potential confounders did not materially change the association. In Cox regression analyses in women, plasma creatine was associated with an increased risk of incident hypertension in the sex and SBP-adjusted model (1.29 [1.11–1.50]; *P* < 0.001). However, this association lost significance after adjustment for age, BMI, eGFR, and urinary albumin excretion (hazard ratio 1.13 [0.96–1.33]; *P* = 0.14). A graphical representation of the sex-based differences in the association between plasma creatine and the risk of incident hypertension is shown in Fig. 3.

### Sensitivity analyses

Firstly, to determine the influence of potential outliers on the found associations, we performed sensitivity analyses after exclusion of patients within the highest or lowest 2.5 percentiles of plasma creatine. Exclusion of these outliers in plasma creatine did not materially change the association between plasma creatine concentration and incident hypertension, as shown in Table S1. Cox regression analyses of plasma creatine with hypertension after exclusion of participants with a baseline SBP between 130 and 139 mmHg and/or a DBP between 80 and 90 mmHg are shown in Table S2. Excluding these participants did not materially change the association between plasma creatine concentration and incident hypertension. Lastly, we investigated the effect of excluding participants with a parent diagnosed with hypertension (Table S3). Exclusion of participants whose parent were diagnosed with hypertension also did not materially change the association between plasma creatine concentration and incident hypertension. A comparison between participants with and without plasma creatine concentration data available is shown in Table S4. The participants differed in sex, parental history of hypertension, total cholesterol, plasma branched chain amino acids and urinary urea excretion (all *P* < 0.05). Each of the aforementioned variables has been adjusted for in the Cox regression analyses.

## DISCUSSION

The cardinal finding of the current study was that higher plasma creatine concentrations were independently associated with an increased risk of incident hypertension. Furthermore, we demonstrated that this association between plasma creatine concentration and the risk of incident hypertension is significantly modified by sex. Higher plasma creatine was independently associated with an increased risk of incident hypertension in men but not in women. Causal pathway analyses demonstrated that this association was not explained by sodium or potassium intake, nor by total and animal-based protein intake reflected by urinary urea and sulfate excretion, respectively.

Creatine is a naturally occurring nitrogenous organic acid that plays an indispensable role in ATP turnover [9]. Through the creatine kinase circuit, creatine and its phosphorylated derivative (phosphocreatine) transfer high-energy phosphate groups from the mitochondria to the cytosol, to regenerate ATP from ADP. Therefore, creatine is especially important in tissues with high and fluctuating energetic demands, most notably skeletal muscles, the brain, and the heart [40,41]. More than 90% of the body's creatine and phosphocreatine is present in muscle, of which in resting muscle cells one-third exists in the form of creatine and two-thirds in the form of phosphocreatine [42]. In clinical medicine, creatine is best known for its continuous, low-grade, nonenzymatic degradation to creatinine, a biomarker for estimating the glomerular filtration rate. The conversion of creatine to creatinine is roughly 1.6–1.7% of the total creatine pool every day, making a continuous replenishment of the creatine pools a necessity [8]. The biochemical synthesis of creatine starts with the enzyme arginine : glycine amidinotransferase (AGAT) converting arginine and glycine into guanidinoacetate, and it is at this step that regulation of endogenous creatine synthesis occurs [13–17]. In humans, highest AGAT activities are present in kidney and pancreas, whereas other tissues, such as brain and testes, express lower activities of AGAT [9,42]. The high expression of AGAT in the kidneys may explain the strong positive association we found between plasma creatine concentration and the estimated glomerular filtration rate. In line with this, an inverse association was found between plasma creatine values and plasma creatinine values. Despite the strong association between kidney function and plasma creatine, the association of plasma creatine with incident hypertension remained significant after adjusting for kidney function in longitudinal models. The second step of creatine synthesis happens preferably in the liver. Guanidinoacetate produced in the kidney is released into the blood stream and, via the γ-aminobutyric-acid-transporter-2 [18], is taken up by the liver. There, guanidinoacetate is methylated by the second enzyme of creatine synthesis, guanidino-acetate-methyl-transferase, using S-adenosyl-methionine as the methyl-group donor [9]. The synthesized creatine is then released into the blood stream from where it is taken up by the target organs.

Products containing high concentrations of creatine, such as meat, fish, and other animal products are usually also relatively high in protein and sodium content [43]. As both protein and salt intake have been linked to blood pressure [44,45], we aimed to elucidate whether the associations of creatine with hypertension is based on protein intake or sodium intake. To assess total protein intake, we used the urinary urea excretion as urea is the principal end product of amino acid degradation, and therefore, parallel to protein intake [46,47]. To assess animal protein intake, we used the urinary sulfate excretion, as meat protein is a rich source of the sulfur-containing amino acids methionine and cysteine and the degradation of these amino acids leads to an increased urinary sulfate excretion [48,49]. It should be noted, however, that plant protein also contain sulfur-containing amino acids, albeit in lower amounts, and that sulfate can also be present as additive to food and alcoholic beverages, making sulfate an imperfect biomarker for meat intake [50]. In linear regression analyses, we found positive associations of plasma creatine with both urinary urea excretion and urinary sulfate excretion. However, adjustments for urinary urea and sulfate excretion did not materially change the point estimates of the Cox-regression analyses, implicating that the association of creatine with hypertension is independent of dietary protein intake. Likewise, we used the urinary sodium excretion as a biomarker for dietary sodium intake [51–53], and also found a strong positive association between plasma creatine and urinary sodium excretion. However, adjustments for urinary sodium excretion did not materially change the point estimates of the Cox regression analyses, implicating that the association of creatine with hypertension is also independent of dietary sodium intake.

After endogenous synthesis or ingestion, creatine is transported to the tissues through the bloodstream and is subsequently transported intracellularly by the sodium-dependent and chloride-dependent creatine transporter, belonging to the solute carrier group SLC6A8, against a large concentration gradient [54,55]. On the basis of the fact that creatine, as a dipolar Zwitter-ion, is membrane-impermeable, it is assumed that creatine efflux out of cells is negligible and that creatine is in fact intracellularly trapped [9]. Circulating plasma creatine concentrations are influenced by three main mechanisms: the amount of ingested dietary creatine via direct uptake through intestinal creatine transporters [56]; the extent of endogenous creatine synthesis, taking place mainly in kidney and liver [9]; and the magnitude of renal tubular creatine reabsorption by the kidney [57]. Additionally, plasma creatine levels may also be influenced by the fragility of muscle plasma membranes, for example, after intensive exercise, and possibly also by the degree to which creatinine is recycled into creatine by the intestinal flora [9]. Finally, the creatine transporter works as a Na^+^/Cl^−^ cotransporter and its activity depends on the Na^+^/K^+^ ATPase, which removes the Na^+^ co-imported into the cells together with creatine. Thus factors leading to an inhibition of the Na^+^/K^+^ ATPase can theoretically also reduce transmembrane creatine transport [58].

One of the hypotheses underlying the well studied association between creatine kinase and incident hypertension is that a high-plasma creatine kinase content reflects a high kidney creatine kinase content. In turn, this may promote reabsorption of sodium in proximal tubules of the kidney [6], leading to fluid overload contributing to the development of hypertension. In our study, we calculated the fractional reabsorption of sodium, defined as 1 − the fractional excretion, as a marker for renal tubular sodium reabsorption. However, in linear regression analyses, we found no association between plasma creatine concentrations and renal tubular sodium reabsorption.

Although we are unable to determine the underlying mechanisms of the found associations, it is noteworthy that we found that higher plasma creatine concentrations were associated with higher total cholesterol, lower HDL-cholesterol, and higher triglycerides. In addition, higher plasma creatine concentrations were also associated with higher glucose and insulin concentrations. Interestingly, these associations were both stronger in men than in women. Furthermore, plasma creatine was also associated with total BCAA, which is considered to be a marker of intestinal dysbiosis, as well as of mitochondrial dysfunction and insulin resistance [59–61]. However, the association of creatine with incident hypertension was not modified by plasma levels of total BCAA, suggesting that BCAA do not play a key role in the causal pathway of creatine and hypertension development.

Noteworthy strengths of this study were the size of the study, the long-term follow-up, and the extensive data collection, allowing for the adjustment for a wide variety of potential confounders. Several limitations of this study need to be addressed. Firstly, because of the observational design of this study, it remains to be determined whether the found relationship between plasma creatine and incident hypertension is causal or associative. Second, we were neither able to determine the underlying mechanisms of the found associations nor were we able to explain why the association between plasma creatine and hypertension is modified by sex. Third, the procedure to enrich the PREVEND cohort with participants with an urinary albumin concentration at least 10 mg/l may have increased the number of participants with hypertension at the baseline of the current study. However, the prevalence at baseline of the current study (34.5%) was not much higher than the prevalence of 31.5%, which has been reported for the global adult general population [62]. Fourth, during recruitment of the PREVEND study, there was a higher degree of nonconsent in the urinary albumin concentration less than 10 mg/l group, which may have induced bias we cannot adjust for.

Also, despite adjusting for many potential confounders, the possibility of residual confounding remains. Furthermore, considering that the selection of participants into the PREVEND cohort was based on urinary albumin concentration (with enrichment for participants with a UAC >10 mg/l), it may not be possible to extrapolate the results to the general population as the prevalence rates of various cardiovascular risk factors may not be comparable with those in the general population. Similarly, the age criteria excluded children and elderly, further preventing extrapolation to the general population. Lastly, we noticed that the amount of participants using insulin during recruitment was higher than to be expected from a Western country, which is consistent with the notion that the current study is not a direct reflection of the general population [63].

In conclusion, we demonstrated that plasma creatine concentrations are lower in men than in women. In time-to-event analyses, higher plasma creatine is associated with an increased risk of incident hypertension, independent of potential confounders. Sex-stratified analyses demonstrated that higher plasma creatine was associated with an increased risk of incident hypertension in men but not in women. These findings implicate a potential role of creatine in the pathophysiology of hypertension. Future studies are warranted to define in more detail the underlying mechanisms for these sex-based differences of the association between plasma creatine and incident hypertension.

## ACKNOWLEDGEMENTS

The Dutch Kidney Foundation supported the infrastructure of the PREVEND program from 1997 to 2003 (Grant E.033). The University Medical Center Groningen supported the infrastructure from 2003 to 2006. Dade Behring, Ausam, Roche, and Abbott financed laboratory equipment and reagents. The Dutch Heart Foundation supported studies on lipid metabolism from 2001 to 2005.

Author contributions: all authors have substantially contributed to the manuscript design and/or revision and have approved this final version of the work. The authors’ responsibilities were as follows: A.P. conducted the literature search, analyzed the data, and created the figures. A.P. and S.J.L.B. drafted the initial manuscript. A.P., D.K., J.C.S., S.S., F.A.V., D.G., I.P.K., E.G., M.A.G., T.W., R.P.F.D., C.F.M.F., S.J.L.B. revised and edited the manuscript. M.A.C. and E.G. acquired the creatine data.

Data Share Statement: data described in the manuscript, code book, and analytic code will be made available upon request of the editor.

### Conflicts of interest

The University Medical Center Groningen received research support from Labcorp in the form of laboratory assessments to R.P.F.D. and S.J.L.B., E.G. and M.A.C. are employees of Labcorp.

S.S. received funding from the European Union's Horizon 2020 research and innovation program under the Marie Sklodowska-Curie grant agreement 754425.

## Supplementary Material

Supplemental Digital Content
