# Supplementary material for: Plasma creatine concentration is associated with incident hypertension in a cohort enriched for the presence of high urinary albumin concentration: the Prevention of Renal and Vascular Endstage Disease study
Source: J Hypertens. 2021 Aug 9;40(2):229–39. doi: 10.1097/HJH.0000000000002996 (PMC8728759; doi:10.1097/HJH.0000000000002996)
Supplement: Supplemental Digital Content [file jhype-40-229-s001.docx]

**Supplementary data to
Plasma Creatine Concentration is Associated With Incident Hypertension in a Cohort Enriched for the Presence of High Urinary Albumin Concentration: The PREVEND Study**


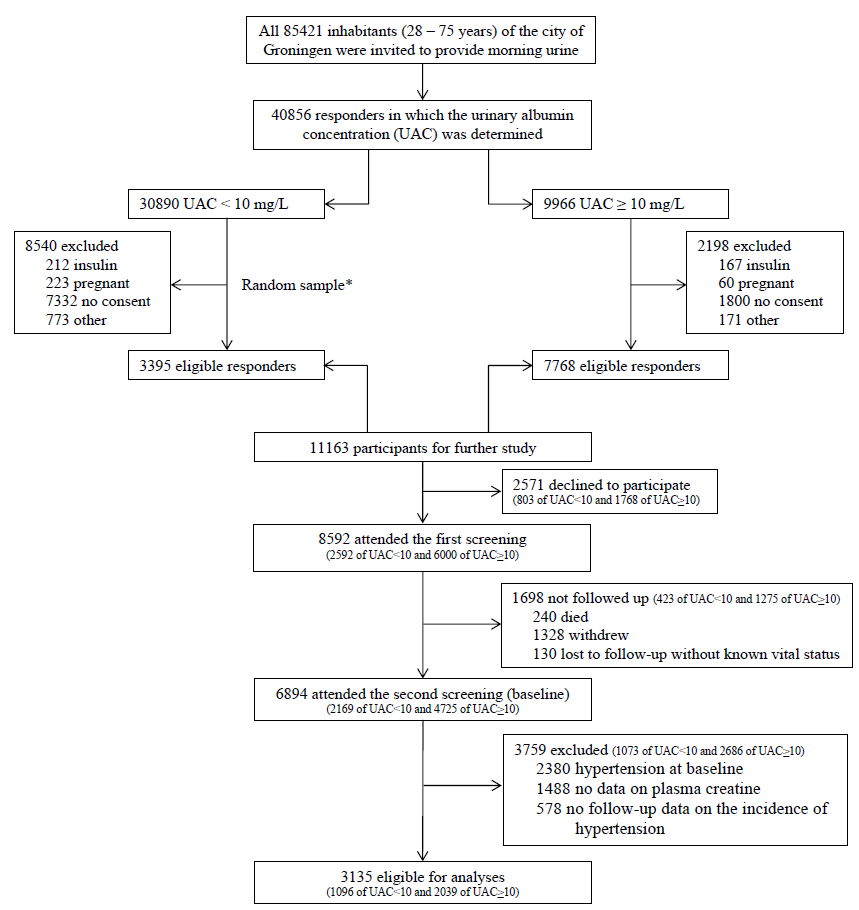


**Figure S1.** Flow of the participants through the study. * Out of 30,890 participants with an UAC < 10 mg/L, 22,350 were eligible responders. Out of these eligible responders, a random sample was selected in order to obtain a total cohort size of roughly 10,000, taking into account a 15% non-participation rate.

| **Table S1.** Prospective associations of plasma creatine with risk of incident hypertension after exclusion the highest and lowest 2.5 percentiles. | | | | | | |
| --- | --- | --- | --- | --- | --- | --- |
|  | **Total cohort** | | **Males** | | **Females** | |
|  | **Per doubling** | | **Per doubling** | | **Per doubling** | |
|  | **HR [95% CI]** | **P-value** | **HR [95% CI]** | **P-value** | **HR [95% CI]** | **P-value** |
| **Model 1** | 1.41 [1.26 – 1.58] | <0.001 | 1.49 [1.28 – 1.73] | <0.001 | 1.30 [1.09 – 1.55] | 0.004 |
| **Model 2** | 1.24 [1.10 – 1.40] | <0.001 | 1.28 [1.10 – 1.50] | 0.002 | 1.17 [0.98 – 1.41] | 0.09 |
| **Model 3** | 1.22 [1.08 – 1.37] | 0.002 | 1.27 [1.08 – 1.49] | 0.004 | 1.13 [0.94 – 1.37] | 0.20 |
| **Model 4** | 1.22 [1.08 – 1.38] | 0.001 | 1.28 [1.09 – 1.50] | 0.003 | 1.13 [0.94 – 1.38] | 0.20 |
| **Model 5** | 1.29 [1.08 – 1.54] | 0.005 | 1.24 [1.05 – 1.46] | 0.01 | 1.12 [0.93 – 1.36] | 0.23 |
| **Model 6** | 1.18 [1.04 – 1.33] | 0.01 | 1.20 [1.02 – 1.41] | 0.03 | 1.12 [0.92 – 1.35] | 0.25 |
| Model 1: adjusted for sex (only for total cohort) and systolic blood pressure. Model 2: as model 1, additionally adjusted for age and BMI. Model 3; as model 2, additionally adjusted for eGFR and urinary albumin excretion. Model 4, as model 3, additionally adjusted for parental history of hypertension and NT-proBNP. Model 5, as model 3, additionally adjusted for total cholesterol, HDL cholesterol, triglycerides and usage of lipid-lowering drugs. Model 6, as model 3, additionally adjusted for smoking and alcohol intake. | | | | | | |

| **Table S2.** Prospective associations of plasma creatine with risk of incident hypertension after exclusion of participants with a systolic blood pressure between 130 and 139 mmHg and/or a diastolic blood pressure between 80 and 90 mmHg | | | | | | |
| --- | --- | --- | --- | --- | --- | --- |
|  | **Total cohort** | | **Males** | | **Females** | |
|  | **Per doubling** | | **Per doubling** | | **Per doubling** | |
|  | **HR [95% CI]** | **P-value** | **HR [95% CI]** | **P-value** | **HR [95% CI]** | **P-value** |
| **Model 1** | 1.39 [1.26 – 1.54] | <0.001 | 1.47 [1.29 – 1.69] | <0.001 | 1.29 [1.10 – 1.51] | 0.002 |
| **Model 2** | 1.22 [1.10 – 1.36] | <0.001 | 1.27 [1.11 – 1.46] | <0.001 | 1.15 [0.98 – 1.36] | 0.09 |
| **Model 3** | 1.21 [1.09 – 1.35] | <0.001 | 1.30 [1.13 – 1.50] | <0.001 | 1.10 [0.93 – 1.30] | 0.28 |
| **Model 4** | 1.22 [1.09 – 1.36] | <0.001 | 1.31 [1.14 – 1.51] | <0.001 | 1.10 [0.93 – 1.30] | 0.28 |
| **Model 5** | 1.27 [1.08 – 1.48] | 0.003 | 1.26 [1.09 – 1.46] | 0.002 | 1.08 [0.92 – 1.28] | 0.35 |
| **Model 6** | 1.17 [1.05 – 1.30] | 0.005 | 1.22 [1.05 – 141] | 0.008 | 1.08 [0.91 – 1.27] | 0.37 |
| Model 1: adjusted for sex (only for total cohort) and systolic blood pressure. Model 2: as model 1, additionally adjusted for age and BMI. Model 3; as model 2, additionally adjusted for eGFR and urinary albumin excretion. Model 4, as model 3, additionally adjusted for parental history of hypertension and NT-proBNP. Model 5, as model 3, additionally adjusted for total cholesterol, HDL cholesterol, triglycerides and usage of lipid-lowering drugs. Model 6, as model 3, additionally adjusted for smoking and alcohol intake. | | | | | | |

| **Table S3.** Prospective associations of plasma creatine with risk of incident hypertension after exclusion of participants with a parental history of hypertension . | | | | | | |
| --- | --- | --- | --- | --- | --- | --- |
|  | **Total cohort** | | **Males** | | **Females** | |
|  | **Per doubling** | | **Per doubling** | | **Per doubling** | |
|  | **HR [95% CI]** | **P-value** | **HR [95% CI]** | **P-value** | **HR [95% CI]** | **P-value** |
| **Model 1** | 1.37 [1.22 – 1.54] | <0.001 | 1.48 [1.27 – 1.71] | <0.001 | 1.20 [0.99 – 1.45] | 0.06 |
| **Model 2** | 1.18 [1.05 – 1.33] | 0.005 | 1.24 [1.07 – 1.45] | 0.005 | 1.09 [0.89 – 1.32] | 0.40 |
| **Model 3** | 1.18 [1.04 – 1.33] | 0.008 | 1.26 [1.08 – 1.47] | 0.003 | 1.06 [0.86 – 1.30] | 0.57 |
| **Model 4** | 1.18 [1.03 – 1.35] | 0.02 | 1.27 [1.06 – 1.52] | 0.009 | 1.06 [0.85 – 1.32] | 0.60 |
| **Model 5** | 1.23 [1.03 – 1.47] | 0.02 | 1.23 [1.05 – 1.44] | 0.01 | 1.06 [0.86 – 1.30] | 0.57 |
| **Model 6** | 1.15 [1.02 – 1.30] | 0.03 | 1.20 [1.02 – 1.41] | 0.03 | 1.05 [0.86 – 1.29] | 0.63 |
| Model 1: adjusted for sex (only for total cohort) and systolic blood pressure. Model 2: as model 1, additionally adjusted for age and BMI. Model 3; as model 2, additionally adjusted for eGFR and urinary albumin excretion. Model 4, as model 3, additionally adjusted for and NT-proBNP. Model 5, as model 3, additionally adjusted for total cholesterol, HDL cholesterol, triglycerides and usage of lipid-lowering drugs. Model 6, as model 3, additionally adjusted for smoking and alcohol intake. | | | | | | |

| **Table S4. Baseline clinical and laboratory characteristics in participants with and without creatine data available** | | | |
| --- | --- | --- | --- |
| **Variables** | **No creatine available** | **Creatine available** | **P-value** |
| Age, years | 50.0 ± 10.6 | 49.3 ± 10.2 | 0.12 |
| Sex, n male (%) | 433 (54) | 1441 (46) | <0.001 |
| BMI, kg/m^2^ | 25.9 ± 4.0 | 25.7 ± 3.8 | 0.18 |
| Systolic blood pressure, mm Hg | 118 ± 11 | 118 ± 11 | 0.08 |
| Diastolic blood pressure, mm Hg | 71 ± 7 | 70 ± 7 | 0.38 |
| Pulse rate, bpm | 68 ± 9 | 68 ± 9 | 0.83 |
| NT-proBNP, ng/L | 34 [18, 61] | 35.0 [18, 62] | 0.50 |
| eGFR, ml/min/1.73m^2^ | 96.4 ± 13.6 | 97.2 ± 14.3 | 0.15 |
| Parental history of hypertension, n (%) | 206 (28) | 952 (33) | 0.03 |
| Smoking status, current n (%) | 254 (32) | 930 (30) | 0.28 |
| Alcohol intake, n yes (%) | 626 (78) | 2440 (78) | 0.92 |
| Lipid lowering drugs, n (%) | 31 (4) | 94 (4) | 0.41 |
| Total cholesterol, mmol/L | 5.46 ± 1.06 | 5.36 ± 1.03 | 0.01 |
| HDL cholesterol, mmol/L | 1.28 ± 0.32 | 1.28 ± 0.30 | 0.76 |
| Triglycerides, mmol/L | 1.04 [0.75, 1.53] | 1.03 [0.76, 1.47] | 0.74 |
| Glucose, mmol/L | 4.7 [4.3, 5.1] | 4.7 [4.4, 5.1] | 0.53 |
| Insulin, mU/L | 7.5 [5.3, 10.9] | 7.3 [5.3, 10.5] | 0.59 |
| Plasma branched chain amino acids, μmol/L | 383.8 ± 77.3 | 370.74 ± 69.8 | <0.001 |
| Urinary albumin excretion, mg/24-h | 7.9 [5.9, 12.1] | 7.8 [5.8, 12.3] | 0.81 |
| Urinary sodium excretion, mmol/24-h | 148 ± 54 | 145 ± 55 | 0.21 |
| Urinary potassium excretion, mmol/24-h | 70.7 ± 21.9 | 70.4 ± 22.2 | 0.70 |
| Urinary urea excretion, mmol/24-h | 381 ± 113 | 366 ± 113 | 0.001 |
| Urinary sulfate excretion, mmol/24-h | 17.3 ± 6.6 | 17.4 ± 17.8 | 0.91 |
| Sodium reabsorption, % | 99.4 ± 0.2 | 99.4 ± 0.2 | 0.63 |
| Results were expressed as mean ± standard deviation (SD), median [interquartile range], or number (percentage) for normally distributed, skewed, and categorical data, respectively. | | | |
